# Learning and Unlearning of Pain

**DOI:** 10.3390/biomedicines6020067

**Published:** 2018-06-05

**Authors:** Larissa Cordier, Martin Diers

**Affiliations:** Department of Psychosomatic Medicine and Psychotherapy, LWL University Hospital, Ruhr University Bochum, 44791 Bochum, Germany; larissa.cordier@rub.de

**Keywords:** chronic pain, learning, classical conditioning, operant conditioning, observational learning, extinction, operant behavioral treatment, cognitive behavioral treatment

## Abstract

This review provides an overview of learning mechanisms and memory aspects for the development of chronic pain. Pain can be influenced in important ways by an individual’s personality, by family, and by the sociocultural environment in which they live. Therefore, learning mechanisms can explain why pain experience and pain behavior can increase or decrease. Linking pain with positive consequences or removing negative consequences can contribute significantly to the chronification of pain. We will provide an overview of treatment options that use the characteristics of extinction. Operant extinction training and cognitive behavioral approaches show promising results for the treatment of chronic pain.

## 1. Introduction

Learning mechanisms are a basic concept in behavioral medicine. There are different mechanisms involved in learning, such as classical and operant conditioning, extinction, habituation, and sensitization. Classical conditioning is a learning procedure in which a biological potent stimulus is paired with a former neutral stimulus. As a result of the pairing, the neutral stimulus elects a response without the biological potent stimulus. Ivan Pavlov, who conditioned a salivation response to the sound of a bell, first described this learning procedure [1]. Whenever he fed his dog, he rang a bell and gave him food. After some time, the dogs salivation was present just by ringing the bell without presenting the food. Another learning procedure is operant conditioning. This procedure is characterized by the fact that the behavior is modified by reinforcement or punishment. During this learning process, an association is made between a behavior and a consequence for that behavior [2]. For example, when someone trains a dog to fetch a ball, he praises him and pats him on the head whenever the dog performs the correct behavior. But if the dog is grubbing a flower bed, he will be punished to reduce this kind of behavior. The question arises as to whether it is possible to reverse the learning process and unlearn a behavior. This process is called extinction and it can be used to unlearn classical or operant conditioned behavior [3]. If Pavlov’s dog hears a bell but no food occurs, the association between these stimuli will weaken, or if someone forgets to praise and pat the dog, he will show less of the desired behavior. However, extinction is not the same as oblivion.

Learning can also be gained through observation and replication of other behavior. Albert Bandura [4] adds two ideas to the principals of behavioral learning theories: 1. mediating processes occur between stimuli and responses; and 2. behavior is learned from the environment through the process of observational learning. In a famous study by Bandura [5], children observed the behavior of an adult dealing with a doll. After the children were left alone with the doll, they imitated the seen behavior of the adults.

Is it possible to adapt to stimuli without explicitly responding to them? According to Groves and Thompson, these learning mechanisms rely on two processes: habituation and sensitization [6]. Habituation occurs if someone no longer notices a stimulus that is repeatedly presented without a reward, punishment, or change in intensity [7]. For example, someone lives in an apartment next to a train station. At the beginning, he perceives the train noises as annoying and hears every motion of the train, but after some time, the person adapts to the noises and will not hear them anymore. The opposite of habituation is sensitization. Sensitization refers to the increase in the strength of a reaction with repeated presentation of the same stimulus. Even though both processes are not conscious, they interact to help people understand their surroundings by strengthening some stimuli and diminishing others [6,8].

However, it has to be noted that all learning mechanisms and behaviors described above can be influenced in important ways by an individual’s personality, family, and the sociocultural environment.

### Pain

Pain is an important evolutionary response signaling danger to the body and triggering protective responses. There is a distinction between acute and chronic pain. Acute pain is usually the reaction to stimulation, has a direct cause, and fulfills an adaptive signal function, whereas chronic pain (duration of at least 3–6 months and/or exceeding the usual healing time for acute injuries) conditions often have no direct cause of pain. After chronification, the pain has lost its basic positive function and becomes an independent disease, which is associated with significant limitations and requires special treatment [9]. If someone suffers from a chronic disease, such as chronic pain, this chronic disease is often associated with a great burden. This burden can result from the disease itself, but also from the associated diagnostic and medical therapeutic measures. The uncertainty about the future of living with this chronic disease can be a particularly strong stressor [9].

## 2. Learning Mechanisms

### 2.1. Operant Learning

The most influential psychological model for the chronification of pain was the assumption of Fordyce [10,11]. Chronic pain can get worse by pain behavior. Fordyce postulated that acute pain behavior, such as moaning or hobbling, under the control of an external amplifiercould strengthen the pain and contribute to its chronification. The learning mechanisms behind this process are positive reinforcement (e.g., attention or the expression of pity), negative reinforcement of pain behavior (e.g., adopting a pain relieving posture), and a lack of reinforcement of healthy behavior (e.g., physical activity). These learning processes can maintain chronic pain, even in the absence of a nociceptive influx. Linking pain with positive consequences or removing negative consequences leads to increased pain behavior at all levels (e.g., work, leisure, and family) and can contribute significantly to the chronification of pain [10,11,12].

Pain behavior, originally induced by nociceptive processes, can occur because of learned environmental contingencies. This model has generated a lot of research; not only have Fordyce’s original assumptions been confirmed, but research was also able to prove that pain sensation and physiological processes of pain processing are operant conditioned. Caregivers, who have a high amplifier potential, play an important role. The reaction towards people who suffer from pain can be divided into three different types: those who reinforce the pain (e.g., expression of compassion, solicitous responses, attention), those who try to distract them from pain (e.g., taking a walk), and those who ignore them (e.g., go out of the room) [13,14]. In a test situation, the pain behavior of chronic pain patients depended on the presence or absence and the reinforcement pattern of their significant other. When the significant other showed comforting and caring behavior towards the chronic pain patient, the pain increased in contrast to a situation without the significant other [13]. Findings by Prenevost and Reme [15] also underline the importance of a significant other on pain coping strategies. During the process of dealing with pain, the interaction between the significant other and the pain patient is an important underlying factor. The significant other is also relevant for the unlearning of pain and in handling its life changing consequences [16,17]. 

Equally important are conditioning processes of taking pain medication. Patients often hear from their doctors or well-meaning family members that they should take painkillers if the pain is really strong and they ‘need’ the medication. Patients learn to associate high perceived pain with medication intake. The pain might be reduced in the beginning, but along the way the amount of medication and the frequency of medication intake will increase, leading to medication abuse or dependency. For the effectiveness of a certain pain medication, however, a constant plasma level might be needed. Thus, both behavioral therapists and physicians/pharmacologists recommend a time-contingent medication intake, instead of a pain-contingent medication intake. This means the medication should be taken at fixed times of the day, independent from the pain intensity [18]. 

The negative reinforcement of the activity level is also an important process in the development of disability. If a specific physical activity—for example, walking—is performed until the occurrence of pain, the patient has to interrupt the activity and rest until the pain decreases. As a result, the patient learns that a reduction of activity reduces pain, leading to lower activity levels and, consequently, muscle atrophy. Therefore, it is necessary that activities are interrupted before they elicit pain. Therefore, the 80% rule is used. Patients should count how many stairs they can climb in one minute. After this, they should only climb 80% of the number of staircases in the same time. This rule can be easily applied to every activity. As a consequence, patients engage in activities without experiencing pain [19].

### 2.2. Respondent Learning and Sensitization

The model of respondent (or classical) conditioning postulates that many previously neutral stimuli are connected to the pain experience from a previous injury. Thus, without the need for a nociceptive input, this association can be coupled with physical reactions and finally trigger pain. From the respondent perspective, muscle tensions formerly elicited from pain are associated with another arbitrarily stimuli. Sitting, standing, bending over, or walking—or even just thinking about these activities—might trigger anxiety and increase muscle tension. This fear of movement, or “kinesiophobia”, as well as the associated fear of pain, are important factors in the development, maintenance, and chronification of pain [12,19,20,21]. In addition, stressful situations can increase the muscle tonus and induce sympathetic activation, which strengthens this process. Many patients report that an acute pain problem becomes chronic if personal stress situations occur simultaneously with pain [19]. Stressful situations can be considered as an additional unconditioned stimulus. Additionally, operant conditioning could occur to a respondent process, and develop avoidance behavior. The avoidance behavior could lead to a reduction of activities and, consequently, to muscle atrophy and disability. Chronic pain patients learn to pay attention to impending pain and increasingly avoid activities resulting in the development of anxiety and depression [19]. The associative linking of neutral stimuli with pain experiences can be a widely branched network of events. This network describes how pain is established and sustained: pain–tension–fear–stress–pain [12].

The repeated presentation of painful stimuli usually leads to habituation, which means a decrease in the reaction to the stimulus. The mediation of sensory information via an applied stimulus increases habituation and diminishes the feeling of surprise, insecurity, and threat [7]. On the contrary, in sensitization, the repeated stimulation leads to an increase in reaction to the stimulus. However, in many chronic pain conditions, sensitization occurs rather than habituation [22]. Being hypersensitive to pain has been referred to as central sensitization. There are two main characteristics for central sensitization, which involve heightened sensitivity to a painful stimulus (hyperalgesia) and the sensation of pain from stimuli (temperature, touch) that do not normally provoke pain (allodynia) [22,23,24].

### 2.3. Observational Learning

Hypotheses about the role of social learning are based on the knowledge that children purchase pain behavior through perception and interpretation of symptoms by watching their parents and other role models [25]. For example, children who observe their parents experiencing pain, and thus avoid pain-related activities, adjust their appreciation of that particular situation and the behavioral consequences. Children basically suffer from symptoms they observe from their parents, and not from those symptoms the parents had as a child themselves, supporting the hypotheses of model learning. Observational learning leads to an accumulation of chronic pain symptoms in families with a chronic pain patient. Thus far, longitudinal studies for observational learning are missing. However, many experiments show that pain tolerance, subjective pain intensity, and non-verbal pain expression to pain stimuli are changing by watching a model handle these situations differently [25]. It is also known that observers of a person to whom painful stimuli were offered exhibit physiological reactions that can be conditioned. For example, pain patients can activate a physical reaction to pain in their significant other, which leads to an increase in pain symptoms and other physical ailments. Investigations show that empathy for pain activates similar brain areas as the direct experience of pain [26].

## 3. Treatment Options

### 3.1. Characteristics of Extinction

Generalization processes happen easily during learning of pain-related behaviors. In contrast, however, the extinction or unlearning of pain is specific to the stimulus and the response, as well as the context during the extinction process [27]. Therefore, it is more difficult for therapists to train a patient to unlearn pain-related behaviors compared with training them to learn these behaviors. During this process, it is not only the erasure of an old memory trace that occurs, but also the learning of a new suppressive process. Changes of memory can fade over time and the new learning processes could be forgotten, a process called recovery. Contrary emotional content as pain and pain behaviors could get stronger over time. The original behavior is a result of generalization to other contexts very resistant to extinction. This could lead to a reactivation of the unlearned memory traces by changing the context. This process is called renewal. Additionally, stressful events (e.g., a new pain episode) could function as an unconditioned stimulus and reactivate the unlearned behavior (reinstatement). Especially for chronic pain patients, reinstatement might be highly relevant as it is likely that new stress and pain episodes occur. Treatments should use many sessions in a short time period and use varying contexts in times with high and low stress. Operant extinction training and cognitive behavioral approaches, which are specifically suited to influence learning and memory processes by modulating alterations in brain function or chemistry, were reported to be successful for the treatment of chronic pain [28,29,30]. 

### 3.2. Operant Extinction Training

It is assumed that patients with high levels of pain behaviors and high interference from pain will benefit from operant extinction training [31]. The goals of this training are as follows: (a) to decrease pain behaviors in an effort to extinguish pain; (b) to increase activity levels and healthy behaviors related to work, leisure time, and family; (c) medication reduction and management; and (d) to change the behavior of significant others. The overall goal is to reduce disability and interference by reducing pain and increasing healthy behaviors, which in turn would increase quality of life. To avoid negative reinforcement learning, medication is switched from a pain-contingent to a fixed-time schedule, where medication is given at certain times of the day. The enhancement of activity and reduction of inactivity and invalidity will be targeted with similar principles. Therefore, weekly schedules and pleasant activities could be used. The focus of patients should be shifted to pleasant activities and not to the pain. To implement this, a significant other of the patient should be involved as a co-therapist, who should help to activate the patient. The significant other learns to reinforce healthy behaviors and to ignore or distract from pain behaviors. Supportive physiotherapy supports the building of muscles. This training has shown its effectiveness in studies training patients with fibromyalgia and chronic back pain [28,29], and it is especially effective in reducing pain behaviors. After an operant behavioral treatment, a shift from an emotional motivational processing of experimental pain to a more sensory discriminative processing was reported [32]. There was a close correlation between the effect of the training and the brain response for the experimental pain stimuli.

### 3.3. Cognitive Behavioral Training

The cognitive-behavioral model of chronic pain emphasizes the role of cognitive, affective, and behavioral factors in the development and maintenance of chronic pain [33]. The cognitive-behavioral training modifies pain-eliciting and maintaining behaviors, cognitions, and emotions to reduce feelings of helplessness and lack of control with the aim of establishing a sense of control over pain. Therefore, a modification of pain triggering and pain maintaining behavior, as well as the involved cognitions and emotions, will be used. In order to do this, patients are taught several techniques to deal with pain episodes, such as cognitive restructuring, pain coping strategies, relaxation and imagery techniques, stress management, and problem solving techniques. It has been shown that cognitive-behavioral pain management is a very effective treatment against chronic pain [29,34,35]. Whereas operant treatment especially reduces pain behaviors and pain intensity, cognitive-behavioral therapy has a special effect on the affective and cognitive aspects of pain [29]. It was suggested that a cognitive-behavioral treatment changes the brain’s processing of pain through an altered cerebral loop between pain signals, emotions, and cognitions, which leads to increased access to executive regions for reappraisal of pain [8]. High catastrophizing thoughts—which were correlated with an increased resting state functional connectivity between the primary somatosensory cortex and the anterior insula—could be reduced by cognitive-behavioral therapy, and could accompany a reduced resting state connectivity between those regions [36]. Because extinction is more difficult than acquisition, principles of extinction training as described above need to be considered [37]. For an overview of randomized controlled trials using operant- or cognitive-behavioral treatments, see Thieme et al. [30]; for a discussion of the potential psychobiological mechanisms, see Diers et al. [32].

## 4. Conclusions and Outlook

The goal of this review was to provide an overview of learning and memory aspects for the development of chronic pain. We provided an overview of some treatment options that use the characteristics of extinction. Operant extinction training and cognitive-behavioral approaches show promising results for the treatment of chronic pain.
